# Author Correction: Effects of route of administration on oxytocin-induced changes in regional cerebral blood flow in humans

**DOI:** 10.1038/s41467-022-29419-w

**Published:** 2022-03-31

**Authors:** D. A. Martins, N. Mazibuko, F. Zelaya, S. Vasilakopoulou, J. Loveridge, A. Oates, S. Maltezos, M. Mehta, S. Wastling, M. Howard, G. McAlonan, D. Murphy, S. C. R. Williams, A. Fotopoulou, U. Schuschnig, Y. Paloyelis

**Affiliations:** 1 Department of Neuroimaging, Institute of Psychiatry, Psychology and Neuroscience, King’s College London, London, UK; 2 South London and Maudsley NHS Foundation Trust, London, UK; 3 Adult Autism and ADHD Service, South London and Maudsley NHS Foundation Trust, London, UK; 4 Institute of Psychiatry, Psychology and Neuroscience, King’s College London, London, UK; 5 Department of Brain Repair and Rehabilitation, Institute of Neurology, University College London, London, UK; 6 Lysholm Department of Neuroradiology, National Hospital for Neurology and Neurosurgery, London, UK; 7 Department of Forensic and Neurodevelopmental Science (SM), Institute of Psychiatry, Psychology and Neuroscience, King’s College London, London, UK; 8 Department of Clinical, Educational and Health Psychology, University College London, London, UK; 9 PARI GmbH, Gräfelfing, Germany

**Keywords:** Drug discovery, Neuroscience

Correction to: *Nature Communications* 10.1038/s41467-020-14845-5, published online 3 March 2020.

In the original version of this Article, there was an error in the calculations of the areas under the curve (AUCs), which systematically overestimated the AUCs for the three methods of oxytocin administration. As a result, Fig. 5, Fig. 6B and C, and Supplementary Table 2 and Supplementary Table 3, and a part of the Results section, contained errors. Repeating all analyses with the correctly calculated AUCs did not change the pattern of results or the conclusions of the analyses. Below are the revised versions of all Results, Figures and Tables where the error in the AUC calculations could have affected the results.

The original version of this Article contained errors in the Results subsection titled ‘Comparison of pharmacokinetic profiles among treatments’, in which a section of text incorrectly read

‘As intended, the intravenous OT infusion resulted in significantly higher AUC over the observation interval, while the intranasal methods did not differ in terms of AUC (Fig. 6b) (Supplementary Table 2) (repeated measures One-way ANOVA: F(1.15, 24.24) = 20.47, *p* < 0.001, η2*p* = 0.7460; post-hoc tests: Spray vs Nebulizer—*p* = 0.1061; Spray vs Intravenous—*p* = 0.0053; Nebulizer vs Intravenous—*p* < 0.0001). There were no significant differences in the absolute bioavailability of OT absorbed to the plasma between the standard nasal spray and PARI SINUS nebulizer (Fig. 6c) (Supplementary Table 2) (paired t-test: T(15) = 1.662, *p* = 0.129, *d* = 0.416)’.

The correct version reads

‘As intended, the intravenous OT infusion resulted in significantly higher AUC over the observation interval, while the intranasal methods did not differ in terms of AUC (Fig. 6b) (Supplementary Table 2) (repeated measures One-way ANOVA: F(1.15,24.24) = 35.80, *p* < 0.001, η2*p* = 0.705; post-hoc tests: Spray vs Nebulizer—*p* = 0.980; Spray vs Intravenous—*p* < 0.001; Nebulizer vs Intravenous—*p* < 0.001). There were no significant differences in the absolute bioavailability of OT absorbed to the plasma between the standard nasal spray and PARI SINUS nebulizer (Fig. 6c) (Supplementary Table 2) (paired t-test: T(15) = 0.796, *p* = 0.438, *d* = 0.199)’.

The original version of Fig. 5 contained errors in data points in all four panels owing to the incorrect AUC calculations.

The correct version of Fig. 5 is:

which replaces the previous incorrect version:

The original version of Fig. 6 contained errors in the data points shown in panels Fig. 6B and C, as well as the reported *p* values in Fig. 6B and the *y* axis label in Fig. 6B owing to the incorrect AUC calculations.

The correct version of Fig. 6 is:

which replaces the previous incorrect version:

This has been corrected in both the PDF and HTML versions of the Article.

The original version of the Supplementary Information associated with this Article contained errors in Supplementary Table 2 (columns labelled ‘AUC (pg.ml-1.h-1)’) and Supplementary Table 3 (columns labelled ‘Absolute bioavailability (%)’). The HTML has been updated to include a corrected version of the Supplementary Information; the original incorrect versions of these Tables can be found as Supplementary Information associated with this Correction.

The original version of the Source Data File associated with this Article contained errors in Source Data associated with Fig. 5 and 6. The HTML has been updated to include a corrected version of the Source Data File; the original incorrect source data associated with those figures can be found as a Source Data File associated with this Correction.

## Supplementary information

Updated Supplementary Information

Updated Source Data

Incorrect Source Data

Incorrect SI File

