# Supplementary material for: Effects of route of administration on oxytocin-induced changes in regional cerebral blood flow in humans
Source: Nat Commun. 2020 Mar 3;11:1160. doi: 10.1038/s41467-020-14845-5 (PMC7054359; doi:10.1038/s41467-020-14845-5)
Supplement: Supplementary file 3 — Reporting Summary [file 41467_2020_14845_MOESM3_ESM.pdf]

nature research

Corresponding author(s): Yannis Paloyelis

Last updated by author(s): 2020/01/12

Reporting Summary

Nature Research wishes to improve the reproducibility of the work that we publish. This form provides structure for consistency and transparency in reporting. For further information on Nature Research policies, see Authors & Referees and the Editorial Policy Checklist .

Statistics

For all statistical analyses, confirm that the following items are present in the figure legend, table legend, main text, or Methods section.

n/a	Confirmed	
☐	☒	The exact sample size (*n*) for each experimental group/condition, given as a discrete number and unit of measurement
☐	☒	A statement on whether measurements were taken from distinct samples or whether the same sample was measured repeatedly
☐	☒	The statistical test(s) used AND whether they are one- or two-sided<br>*Only common tests should be described solely by name; describe more complex techniques in the Methods section.*
☐	☒	A description of all covariates tested
☐	☒	A description of any assumptions or corrections, such as tests of normality and adjustment for multiple comparisons
☐	☒	A full description of the statistical parameters including central tendency (e.g. means) or other basic estimates (e.g. regression coefficient) AND variation (e.g. standard deviation) or associated estimates of uncertainty (e.g. confidence intervals)
☐	☒	For null hypothesis testing, the test statistic (e.g. *F*, *t*, *r*) with confidence intervals, effect sizes, degrees of freedom and *P* value noted<br>*Give P values as exact values whenever suitable.*
☒	☐	For Bayesian analysis, information on the choice of priors and Markov chain Monte Carlo settings
☒	☐	For hierarchical and complex designs, identification of the appropriate level for tests and full reporting of outcomes
☐	☒	Estimates of effect sizes (e.g. Cohen's *d*, Pearson's *r*), indicating how they were calculated

Our web collection on statistics for biologists contains articles on many of the points above.

Software and code

Policy information about availability of computer code

Data collection	No software was used
Data analysis	ASAP (Automatic Software for ASL processing) toolbox (version 2.0); FMRIB Software Library (FSL); SPM12 software; Kubios HRV analysis software (MATLAB, version 2 beta, Kuopio, Finland); SPSS 24 (http://www-01.ibm.com/software/uk/analytics/spss/); ggplot2 package from R

For manuscripts utilizing custom algorithms or software that are central to the research but not yet described in published literature, software must be made available to editors/reviewers. We strongly encourage code deposition in a community repository (e.g. GitHub). See the Nature Research guidelines for submitting code & software for further information.

Data

Policy information about availability of data

All manuscripts must include a data availability statement. This statement should provide the following information, where applicable:

- Accession codes, unique identifiers, or web links for publicly available datasets
- A list of figures that have associated raw data
- A description of any restrictions on data availability

Data can be accessed from the corresponding author upon reasonable request. A reporting summary for this Article is available as a Supplementary Information file.

Field-specific reporting

Please select the one below that is the best fit for your research. If you are not sure, read the appropriate sections before making your selection.

Behavioural & social sciences study design

All studies must disclose on these points even when the disclosure is negative.

Study description	Double-blind, placebo-controlled, triple-dummy, crossover design
Research sample	We recruited 17 healthy male adult volunteers (mean age 24.5, SD = 5, range 19-34 years). We determined sample size based on our previous validation study demonstrating that N=16 per group was sufficient to quantify standard nasal spray OT-induced changes in rCBF in a between-subjects design
Sampling strategy	Participants were recruited using the King's College London online circular. We screened participants for psychiatric conditions using the Symptom Checklist-90-Revised and the Beck Depression Inventory-II questionnaires. Participants were not taking any prescribed drugs, did not have a history of drug abuse and tested negative on a urine panel screening test for recreational drugs, consumed <28 units of alcohol per week and <5 cigarettes per day. We instructed participants to abstain from alcohol and heavy exercise for 24 hours and from any beverage or food for 2 hours before scanning.
Data collection	We employed a double-blind, placebo-controlled, triple-dummy, crossover design. Participants visited our centre for 4 experimental sessions spaced 8.90 days apart on average (SD = 5.65, range: 5-28 days). In each session, participants received treatment via all three administration routes, in one of two fixed sequences: either nebulizer/intravenous infusion/standard nasal spray, or standard nasal spray/intravenous infusion/nebulizer. Participants were randomly allocated to a treatment order (i.e. a specific plan regarding which route delivered the active drug in each experimental session) that was determined using a Latin square design. Unbeknown to the participants, the first treatment administration method in each session always contained placebo. while intranasal (spray or nebulizer) OT was only delivered with the third treatment administration. After drug administration, participants were guided to the MRI scanner, where eight pulsed continuous arterial spin labeling scans (each lasting approx. eight minutes) where acquired, spanning 15-104 minutes post-dosing
Timing	Data collection started at 09/06/2015 and finished at 20/01/2016
Data exclusions	One participant did not complete one of the four visits and for this reason was excluded from all analyses.
Non-participation	One participant did not complete one of the four visits and for this reason was excluded from all analyses.
Randomization	Participants were randomly allocated to a treatment order (i.e. a specific plan regarding which route delivered the active drug in each experimental session) that was determined using a Latin square design.

Reporting for specific materials, systems and methods

We require information from authors about some types of materials, experimental systems and methods used in many studies. Here, indicate whether each material, system or method listed is relevant to your study. If you are not sure if a list item applies to your research, read the appropriate section before selecting a response.

Materials & experimental systems

n/a	Involved in the study	
☐	☐	Antibodies
☐	☐	Eukaryotic cell lines
☐	☐	Palaeontology
☐	☐	Animals and other organisms
☐	☒	Human research participants
☐	☐	Clinical data

Methods

n/a	Involved in the study	
☐	☐	ChIP-seq
☐	☐	Flow cytometry
☐	☒	MRI-based neuroimaging

Antibodies

Antibodies used	*Describe all antibodies used in the study; as applicable, provide supplier name, catalog number, clone name, and lot number.*
Validation	*Describe the validation of each primary antibody for the species and application, noting any validation statements on the manufacturer's website, relevant citations, antibody profiles in online databases, or data provided in the manuscript.*

Eukaryotic cell lines

Policy information about cell lines

Cell line source(s)	*State the source of each cell line used.*
Authentication	*Describe the authentication procedures for each cell line used OR declare that none of the cell lines used were authenticated.*

Mycoplasma contamination | *Confirm that all cell lines tested negative for mycoplasma contamination OR describe the results of the testing for mycoplasma contamination OR declare that the cell lines were not tested for mycoplasma contamination.*

Commonly misidentified lines (See ICLAC register) | *Name any commonly misidentified cell lines used in the study and provide a rationale for their use.*

Palaeontology

Specimen provenance | *Provide provenance information for specimens and describe permits that were obtained for the work (including the name of the issuing authority, the date of issue, and any identifying information).*

Specimen deposition | *Indicate where the specimens have been deposited to permit free access by other researchers.*

Dating methods | *If new dates are provided, describe how they were obtained (e.g. collection, storage, sample pretreatment and measurement), where they were obtained (i.e. lab name), the calibration program and the protocol for quality assurance OR state that no new dates are provided.*

☐ Tick this box to confirm that the raw and calibrated dates are available in the paper or in Supplementary Information.

Animals and other organisms

Policy information about studies involving animals; ARRIVE guidelines recommended for reporting animal research

Laboratory animals | *For laboratory animals, report species, strain, sex and age OR state that the study did not involve laboratory animals.*

Wild animals | *Provide details on animals observed in or captured in the field; report species, sex and age where possible. Describe how animals were caught and transported and what happened to captive animals after the study (if killed, explain why and describe method; if released, say where and when) OR state that the study did not involve wild animals.*

Field-collected samples | *For laboratory work with field-collected samples, describe all relevant parameters such as housing, maintenance, temperature, photoperiod and end-of-experiment protocol OR state that the study did not involve samples collected from the field.*

Ethics oversight | *Identify the organization(s) that approved or provided guidance on the study protocol, OR state that no ethical approval or guidance was required and explain why not.*

Note that full information on the approval of the study protocol must also be provided in the manuscript.

Human research participants

Policy information about studies involving human research participants

Population characteristics | We recruited 17 healthy male adult volunteers (mean age 24.5, SD = 5, range 19-34 years). One participant did not complete one of the four visits and for this reason was excluded from all analyses.

Recruitment | Internal online circular

Ethics oversight | King's College London Research Ethics Committee (PNM/13/14-163) approved the study.

Note that full information on the approval of the study protocol must also be provided in the manuscript.

Clinical data

Policy information about clinical studies

All manuscripts should comply with the ICMJEguidelines for publication of clinical research and a completedCONSORT checklist must be included with all submissions.

Clinical trial registration | *Provide the trial registration number from ClinicalTrials.gov or an equivalent agency.*

Study protocol | *Note where the full trial protocol can be accessed OR if not available, explain why.*

Data collection | *Describe the settings and locales of data collection, noting the time periods of recruitment and data collection.*

Outcomes | *Describe how you pre-defined primary and secondary outcome measures and how you assessed these measures.*

ChIP-seq

Data deposition

☐ Confirm that both raw and final processed data have been deposited in a public database such as GEO.

☐ Confirm that you have deposited or provided access to graph files (e.g. BED files) for the called peaks.

Data access links
May remain private before publication.

For "Initial submission" or "Revised version" documents, provide reviewer access links. For your "Final submission" document, provide a link to the deposited data.

Files in database submission

Provide a list of all files available in the database submission.

Genome browser session
(e.g. UCSC)

Provide a link to an anonymized genome browser session for "Initial submission" and "Revised version" documents only, to enable peer review. Write "no longer applicable" for "Final submission" documents.

Methodology

Replicates

Describe the experimental replicates, specifying number, type and replicate agreement.

Sequencing depth

Describe the sequencing depth for each experiment, providing the total number of reads, uniquely mapped reads, length of reads and whether they were paired- or single-end.

Antibodies

Describe the antibodies used for the ChIP-seq experiments; as applicable, provide supplier name, catalog number, clone name, and lot number.

Peak calling parameters

Specify the command line program and parameters used for read mapping and peak calling, including the ChIP, control and index files used.

Data quality

Describe the methods used to ensure data quality in full detail, including how many peaks are at FDR 5% and above 5-fold enrichment.

Software

Describe the software used to collect and analyze the ChIP-seq data. For custom code that has been deposited into a community repository, provide accession details.

Flow Cytometry

Plots

Confirm that:

- [ ] The axis labels state the marker and fluorochrome used (e.g. CD4-FITC).
- [ ] The axis scales are clearly visible. Include numbers along axes only for bottom left plot of group (a 'group' is an analysis of identical markers).
- [ ] All plots are contour plots with outliers or pseudocolor plots.
- [ ] A numerical value for number of cells or percentage (with statistics) is provided.

Methodology

Sample preparation

Describe the sample preparation, detailing the biological source of the cells and any tissue processing steps used.

Instrument

Identify the instrument used for data collection, specifying make and model number.

Software

Describe the software used to collect and analyze the flow cytometry data. For custom code that has been deposited into a community repository, provide accession details.

Cell population abundance

Describe the abundance of the relevant cell populations within post-sort fractions, providing details on the purity of the samples and how it was determined.

Gating strategy

Describe the gating strategy used for all relevant experiments, specifying the preliminary FSC/SSC gates of the starting cell population, indicating where boundaries between "positive" and "negative" staining cell populations are defined.

- [ ] Tick this box to confirm that a figure exemplifying the gating strategy is provided in the Supplementary Information.

Magnetic resonance imaging

Experimental design

Design type

Resting state

Design specifications

Participants were instructed to lie still and maintain their gaze on a centrally placed fixation cross during scanning. Each arterial spin labeling scan lasted for about 8 minutes.

Behavioral performance measures

We assessed participants' levels of alertness (anchors: alert-drowsy) and excitement (anchors: excited-calm) using visual analogue scales (0-100) at 3 different time-points during the scanning session (the first one immediately before the first scan – around 15 mins post-dosing, the second one immediately after the fourth scan – around 55 mins post-dosing and the last one immediately before the seventh scan – around 92 mins post-dosing) to evaluate subjective drug effects across time.

Acquisition

Imaging type(s) | Arterial Spin Labeling

Field strength | 3 Tesla

Sequence & imaging parameters | We used a 3D pseudo-continuous Arterial Spin Labelling (3D-pCASL) sequence to measure changes in regional Cerebral Blood Flow (rCBF) over 15-120 min post-dosing. Labelling of arterial blood was achieved with a 1525ms train of Hanning shaped RF pulses in the presence of a net magnetic field gradient along the flow direction (the z-axis of the magnet). After a post-labelling delay of 2025ms, a whole brain volume was read using a 3D inter-leaved "stack-of-spirals" Fast Spin Echo readout86, consisting of 8 interleaved spiral arms in the in-plane direction, with 512 points per spiral interleave. TE was 11.088 ms and TR was 5135 ms. 56 slice-partitions of 3mm thickness were defined in the 3D readout. The in-plane FOV was 240×240 mm. The spiral sampling of k-space was re-gridded to a rectangular matrix with an approximate in-plane resolution of 3.6mm. The sequence acquired 5 control-label pairs. Individual CBF maps were computed for each of the perfusion weighted difference images derived from every control-label (C-L) pair, by scaling the difference images against a proton density image acquired at the end of the sequence, using identical readout parameters. This computation was done according to the formula suggested in the recent ASL consensus article87. The sequence uses four background suppression pulses to minimize static tissue signal at the time of image acquisition. We performed eight of these 3D-pCASL sequence acquisitions, the acquisition time of each sequence was 8:20 min. A 3D high-spatial-resolution, Magnetisation Prepared Rapid Acquisition (3D MPRAGE) T1-weighted scan was acquired. Field of view was 270mm, TR/TE/TI = 7.328/3.024/400 ms. The final resolution of the T1-weighted image was 1.1 x 1.1 x 1.2 mm.

Area of acquisition | Whole-brain

Diffusion MRI ☐ Used ☒ Not used

Preprocessing

Preprocessing software | ASAP (Automatic Software for ASL processing) toolbox (version 2.0); FMRIB Software Library (FSL); SPM12 software

Normalization | A multi-step approach was performed for the spatial normalization of the CBF maps computed for each C-L pair to the space of the Montreal Neurological Institute (MNI): (1) co-registration of the proton density image from each sequence to the participant's T1-image after resetting the origin of both images to the anterior commissure. The transformation matrix of this co-registration step was then applied to the CBF map from each C-L pair, to transform the CBF map to the space of the T1-image; (2) unified segmentation of the T1 image; (3) elimination of extra-cerebral signal from the CBF map, by multiplication of the "brain only" binary mask obtained in step [2], with each co-registered CBF map; (4) normalization of the subject's T1 image and the skull-stripped CBF maps to the MNI152 space using the normalisation parameters obtained in step [2]. Finally, we spatially smoothed each normalized CBF map using an 8-mm Gaussian smoothing kernel.

Normalization template | Montreal Neurological Institute (MNI)

Noise and artifact removal | *Describe your procedure(s) for artifact and structured noise removal, specifying motion parameters, tissue signals and physiological signals (heart rate, respiration).*

Volume censoring | None

Statistical modeling & inference

Model type and settings | *Specify type (mass univariate, multivariate, RSA, predictive, etc.) and describe essential details of the model at the first and second levels (e.g. fixed, random or mixed effects; drift or auto-correlation).*

Effect(s) tested | *Define precise effect in terms of the task or stimulus conditions instead of psychological concepts and indicate whether ANOVA or factorial designs were used.*

Specify type of analysis: ☒ Whole brain ☐ ROI-based ☐ Both

Statistic type for inference (See Eklund et al. 2016) | Cluster-wise inference, p<0.05 FWE-corrected, cluster-forming threshold p<0.005

Correction | FWE

Models & analysis

n/a	Involved in the study
☐	☐ Functional and/or effective connectivity
☐	☐ Graph analysis
☐	☐ Multivariate modeling or predictive analysis

Functional and/or effective connectivity

Report the measures of dependence used and the model details (e.g. Pearson correlation, partial correlation, mutual information).

Graph analysis

Report the dependent variable and connectivity measure, specifying weighted graph or binarized graph, subject- or group-level, and the global and/or node summaries used (e.g. clustering coefficient, efficiency, etc.).

Multivariate modeling and predictive analysis

Specify independent variables, features extraction and dimension reduction, model, training and evaluation metrics.
